# Repurposing Candesartan Cilexetil as Antibacterial Agent for MRSA Infection

**DOI:** 10.3389/fmicb.2021.688772

**Published:** 2021-09-13

**Authors:** Lanlan Xu, Pengfei She, Lihua Chen, Shijia Li, Linying Zhou, Zubair Hussain, Yaqian Liu, Yong Wu

**Affiliations:** Department of Laboratory Medicine, The Third Xiangya Hospital, Central South University, Changsha, China

**Keywords:** candesartan cilexetil, *Staphylococcus aureus*, antibacterial, synergistic, resistance, biofilm, persisters, membrane

## Abstract

*Staphylococcus aureus* is an important pathogen causing hospital-acquired infections. Methicillin-resistant *S. aureus* (MRSA), biofilms, and persisters are highly tolerant to traditional antibiotics and make it difficult to treat. Therefore, new antimicrobial agents are urgently needed to treat hard-to-eradicate diseases caused by this bacterium. In this study, candesartan cilexetil (CC), an angiotensin hypertension drug, had strong antimicrobial activity against *S. aureus* with minimal inhibitory concentrations (MICs) and minimal bactericidal concentrations (MBCs) of 8–16 μg/ml and 16–32 μg/ml. CC exhibited limited cytotoxicity and low potential to induce drug resistance. In addition, it showed a synergistic antibacterial effect when combined with gentamicin and tobramycin. The effective concentrations to inhibit MRSA biofilm formation were 16–64 μg/ml, and intractable persisters were killed at 4–8 × MIC. Through the analysis of its mechanism of action, it was evident that the membrane permeability was disrupted as well as the cell structure was damaged. Furthermore, we demonstrated that CC had antibacterial effects *in vivo* in MRSA-infected murine skin abscess models. In conclusion, these results imply that CC might be a potential antibacterial agent for the treatment of *S. aureus*-associated infections.

## Introduction

Multidrug resistant (MDR) bacteria pose a huge therapeutic challenge to the currently available choices of treatment (Le et al., 2020). *Staphylococcus aureus* is a Gram-positive bacterium present in the human skin and mucosae with a pathogenic ability to cause multiple types of infections (Kim W. et al., 2020). *S. aureus* adheres to the surface of medical devices to cause biofilm-associated infections that are more difficult to treat and eradicate (Wi and Patel, 2018). The production of virulence factors and hemolysin aggravates its impact on life and health (Reddinger et al., 2016). What is more serious is that *S. aureus* forms persisters that reduce cellular biosynthesis and metabolism and prevent the entry of certain antibiotics. The persisters within biofilms generally are tolerant to the immune system, which increases the difficulty of treatment (Del Pozo, 2018; Kim et al., 2019).

Therefore, the introduction of novel antimicrobial agents is a pressing priority to control antibiotic-resistant bacteria (Kim W. et al., 2020). One of the strategies to solve this problem is repurposing existing drugs, which are easy to obtain and have known safety with a low price, thereby saving human, financial, and material resources, rather than by discovering new antibacterial classes (Chernov et al., 2019). Some of the repurposed drugs have attracted more attention as they can be used as novel alternatives for antibiotics. Ibuprofen, disulfiram, and carmofur have been found to exert antibacterial effect against *S. aureus* (Oliveira et al., 2019; Thakare et al., 2019).

Candesartan cilexetil (CC) is an angiotensin II receptor antagonist and transformed into an active form of candesartan after intestinal absorption (See and Stirling, 2000). It is clinically used for the treatment of hypertension with oral dose of 8–32 mg once a day (Ardiana et al., 2012). Recently, the efficacy of CC in the treatment of chronic heart failure (CHF) was reported (Fenton and Scott, 2005). Otherwise, CC exhibits potential antiviral effect against Zika virus by inhibiting the synthesis of RNA and protein. Moreover, its antiviral activity extends beyond Zika virus (Loe et al., 2019), but its antibacterial properties have not been reported hitherto. We primarily found that CC had antibacterial potential against *S. aureus* by using high-throughput screening in FDA-approved library. Subsequently, the antibacterial, antibiofilm effect and its synergistic effect in combination with conventional antibiotics were investigated. Antibacterial mechanism with regard to membrane was assessed by SYTOX Green, DISC3(5), scanning electron microscopy (SEM), and transmission electron microscopy (TEM). Finally, we studied the antibacterial activity *in vivo* by subcutaneous abscess model.

## Materials and Methods

### Strains and Cultural Conditions

*Staphylococcus aureus* Newman, ATCC 43300 and RJ-2 (MRSA) were provided by Min Li (Shanghai Jiao Tong University). Mingqiang Qiao (Nankai University) provided the *Pseudomonas aeruginosa* PAO1. *Enterococcus faecalis* ATCC 29212, *Acinetobacter baumannii* ATCC 1195, *Klebsiella pneumoniae* ATCC 700603, *Escherichia coli* ATCC 25922, and *S. aureus* ATCC 29213 were provided by Juncai Luo (Tiandiren Biotech). *Staphylococcus epidermidis* ATCC 12228 was provided by Di Qu (Fudan University). Clinical strains concluding SA 76 (MRSA) and PA47 were collected in the Third Xiangya Hospital, Central South University (She et al., 2020b). All the Gram-positive strains were cultured in tryptic soy broth (TSB) or brain–heart infusion broth (BHI) (Solarbio, Beijing, China). Gram-negative strains were cultured in Luria–Bertani broth (LB) (Solarbio) medium. CC, tobramycin, ciprofloxacin, clindamycin, vancomycin, teicoplanin, and oxacillin were purchased from the MedChem Express, while gentamicin and amikacin were bought from Aladdin (Shanghai, China) and were dissolved in DMSO or deionized water.

### Determination of Minimal Inhibitory Concentration and Minimal Bactericidal Concentration

The detection of MIC was performed by the microbroth dilution method (Wang et al., 2020) with reference to Clinical and Laboratory Standards Institute [CLSI] (2012). The bacterial suspension was adjusted to an equivalent of 0.5 McFarland standard (1.5 × 10^8^ CFU/ml) and then diluted 100 times. Bacterial suspension (50 μl) and equal volume of serial diluted drugs (1–128 μg/ml) were added into a 96-well cell plate (Corning Costar, United States) and incubated for 16–18 h. The bacterial suspension without compound was regarded as negative control. The lowest concentration of drug that inhibited the growth of visible bacteria was deemed as MIC. As for MBC, the suspension with a concentration higher than or equal to MIC was spotted on an agar plate and incubated for 24 h. The lowest concentration where no bacterial colonies grew on the plate was defined as MBC (She et al., 2020a). The concentration of Ca^2+^ was maintained at 50 μg/ml with daptomycin.

### Drug Combination Assay

The checkerboard microdilution method was used to evaluate the synergetic effect between CC and different antibiotics. Concisely, two drugs with a maximum concentration of 2 × MIC were serially diluted and added into a 96-well plate horizontally or vertically. Bacterial cultures were diluted in broth to achieve a final concentration of roughly 5 × 10^5^ CFU/ml. The mixture was incubated for 18 h and detected at OD_630nm_ by a microplate reader (Bio-Rad, United States). The experiment was repeated in triplicate. The synergy was evaluated by the fractional inhibitory concentration index (FICI) according to the following formula:


$$\text{FICI}=\frac{{\text{MIC}}_{\text{A}}\,\left(\mathrm{combination}\right)}{{\text{MIC}}_{\text{A}}\,\left(\mathrm{alone}\right)}+\frac{{\text{MIC}}_{\text{B}}\,\left(\mathrm{combination}\right)}{{\text{MIC}}_{\text{B}}\,\left(\mathrm{alone}\right)}$$

FICI < 0.5 indicates synergism; 0.5 ≤ FICI < 1 indicates partial synergy; FICI = 1 indicates additive effects; 1 < FICI ≤ 4 is irrelevant; FICI > 4 indicates antagonism (Dupieux et al., 2017).

### Time-Kill Assay

Time-kill assay was performed as described previously by Ramchuran et al. (2018). A culture of *S. aureus* was suspended in 10 ml of TSB and incubated with CC at 0.25–2 × MIC. The final bacterial suspension concentration was approximately 1.5 × 10^6^ CFU/ml. Subsequently, samples were harvested, respectively, at the time points of 0, 2, 4, 8, and 12 h for serial dilution, and spotted on blood agar plate to count the bacterial colonies. The colony counting was expressed as CFU/ml. The OD_630nm_ was measured at those time points, and the experiments were repeated three times (Li et al., 2017).

### Persister Killing and Membrane Permeability Assay

Persister cell analysis was based on the technique published by Kim et al. (2019) with slight modifications. Briefly, MRSA was cultured in TSB overnight to the stationary phase to form persisters. The cultures were washed twice and diluted to OD_630nm_ = 0.2. Persisters were subsequently treated with 100 × MIC vancomycin, linezolid, gentamicin, ciprofloxacin, and 10 × MIC CC. After 6 h, the samples were diluted and spotted on a blood agar plate. The colonies were counted after 24-h incubation at 37°C. The same procedure was adopted for the persister killing assay as described above. After washing twice, CC with 2 × MIC, 4 × MIC, and 8 × MIC were added, vancomycin and no drug treatment served as controls. The suspension was diluted and spotted on a plate at 0, 2, 4, and 6 h (Ko et al., 2019). To determine the membrane permeability, the persisters, as mentioned above, were diluted to OD_630nm_ = 0.15 with 5 mM HEPES buffer containing 2 μM SYTOX Green (Thermo Scientific, United States) for 30 min. The HEPES buffer with varying concentrations of CC were incubated with the bacteria/SYTOX Green suspension, while DMSO and melittin were used as controls. The fluorescence intensity was detected by a multimode plate reader (λex/em = 504/523 nm) (PerkinElmer, United States). The results were defined as the fluorescence value minus the background value. All experiments were repeated three times (Kim et al., 2018).

### Biofilm Inhibition and Eradication Assay

The crystal violet (CV) was used to quantify biofilm mass. One hundred microliters of CC with appropriate concentrations (0–64 μg/ml) was added into a 96-well plate and inoculated with 1:50 dilution overnight of bacterial suspension for 24 h. The biofilms were washed and stained with 0.25% CV. Fifteen minutes later, the CV was discarded and washed to remove the remaining dye. Last, 95% ethanol was added to dissolve the dyed CV for 20 min. The absorbance was measured at 570 nm. XTT staining method was obtained following a previous protocol. Concisely, 100 μl of XTT (0.2 mg/ml) and PMS (0.02 mg/ml) (Shanghai Macklin Biochemical Co., Ltd., China) with 1 × PBS were added and incubated for 3 h in the dark. The result was measured at 490 nm using a microplate reader (Gao et al., 2020). In order to assess the viable cells in preformed biofilm, *S. aureus* ATCC 43300 was diluted 1:100 and added to 96-well plates for 24 h to construct mature biofilms. The planktonic bacteria were washed, and 100 μl of the required concentrations of premixed drug was added. After 24 h, the plates were washed, and the biofilms were broken up with tips. Ultimately, the viable cells were counted on the agar plate. The experiments were conducted with biological replicates (Tan et al., 2019).

### Observation of Biofilm Morphology by Confocal Laser Scanning Microscope

The mature biofilm was formed on the sterile glass slides for 24 h. After removing unbound cells, TSB alone or with 64 μg/ml CC was used to treat the biofilm. Another 24 h later, the glass coverslips were washed and stained with a mixture of SYTO9 and PI (Thermo Scientific, United States) for 15 min. The morphology was visualized by CLSM (ZEISS, Germany), and the fluorescence intensity was calculated with ImageJ software (Jiang et al., 2014).

### The Mechanism of Action of Candesartan Cilexetil on *Staphylococcus aureus*

#### Scanning Electron Microscopy and Transmission Electron Microscopy

The culture of *S. aureus* ATCC 43300 in mid-log phase was harvested with 1 × PBS at 3,000 × *g* for 15 min, then treated with 10 × MIC of CC for 1 h. The suspension was centrifuged, and the pellet was collected and fixed with 1 ml of 2.5% glutaraldehyde. The cellar morphology was examined under SEM and TEM (Hitachi, Japan) (Lu et al., 2020).

#### Membrane Permeability Assays and Cytoplasmic Membrane Electrical Potential Measurement

Mid-log-phase cells of *S. aureus* ATCC 43300 were adjusted to OD_630nm_ = 0.05 and incubated with 2 μM SYTOX Green in 5 mM HEPES buffer (PH 7.2, containing 5 mM Glu) in the dark. The suspension was treated with CC (final concentration ranging from 8 to 64 μg/ml), whereas 0.1% DMSO and 10 μg/ml of melittin were used as controls. The fluorescence intensity was monitored for 30 min (λ_ex/em_ = 504/523 nm) (Wu et al., 2019). The membrane potential measurement was slightly different from the above procedure. Briefly, mid-log-phase cells were washed and suspended in 5 mM HEPES buffer, 100 mM KCl, 5 mM Glu, and 2 μM DiSC3(5) (AAT Bioquest, United States) for 5 min. Bithionol (10 μg/ml) was used as control. The fluorescence intensity was measured at the excitation wavelength of 622 nm with emission wavelength of 670 nm (Zhou et al., 2020). The experiments were repeated in triplicate.

#### ATP Determination

*Staphylococcus aureus* ATCC 43300 was cultured for 6–8 h to mid-log phase and washed with 1 × PBS. Next, the bacterial cells were treated with serially diluted CC (8, 16, 32, 64 μg/ml) for 1 h and centrifuged at 10,000 rpm, 4°C, for 5 min. The bacterial precipitates were lysed with lysis buffer and centrifuged. The supernatants were used for intracellular ATP level determination according to the instructions of the Enhanced ATP Assay Kit (Beyotime, China) (Song et al., 2020).

### Resistance Inducing Assay

The continuous passage assay was used to evaluate the appearance of resistance of *S. aureus* to CC. The MICs on the first day were determined as described above. On the next day, 2 μl of bacterial suspension at 0.5 × MIC was diluted 1,000 times and incubated with different concentrations of CC for 16–18 h; afterward, the value of MIC was determined. The experiment was carried out consecutively for 31 days as described above, whereas ciprofloxacin was used as the control (Martin et al., 2020). For one-step resistance screening, *S. aureus* ATCC 43300 and ATCC 29213 were cultured overnight and resuspended in the MH medium at a concentration of OD_630_ = 0.5. One hundred microliters of suspension was smeared on pre-prepared MH plate containing rifampicin, ciprofloxacin, or CC (2 ×, 4 ×), and the number of resistant colonies was calculated after 48 h (Ling et al., 2015).

### Human Blood Cell Hemolysis Assay and CCK-8 Cell Cytotoxicity Test

Human red blood cells (RBCs) were purchased from the Hemo Pharmaceutical and Biological Co (Shanghai, China). Two milliliters of RBCs was centrifuged with 1 × PBS at 1,000 × *g* for 5 min at 4°C and then added to a 96-well plate. The RBCs (final concentration of 5% v/v) were treated with CC at a concentration of 2–64 μg/ml, while the positive and negative controls were 0.1% TritonX-100 and 1% DMSO, respectively. After incubation at 37°C for 1 h, the samples were centrifuged and measured at A_570nm_ (Tan et al., 2020). The results were expressed as HC_50_ (concentration of 50% hemolysis of red blood cells) (Dey et al., 2019). The hemolysis rate was calculated as follows:


$$\mathrm{Hemolysis}\left(\%\right)=\frac{{\text{A}}_{\text{sample}}-{\text{A}}_{1\%\,\mathrm{DMSO}}}{{\text{A}}_{\mathrm{TritonX}-100}-{\text{A}}_{1\%\,\mathrm{DMSO}}}\times 100\%$$

As for cytotoxicity test, human bronchial epithelial cells (HBE), HepG-2, and human skin fibroblast (HSF) cell lines were cultured in Dulbecco’s modified Eagle’s medium (DMEM) or RPMI1640 supplemented with 10% fetal bovine serum (FBS) and 1% double antibiotics. One hundred microliters of log-phase cells was added to the microtiter plate to obtain a final concentration of 3 × 10^3^ (HBE and hepG-2) and 7 × 10^3^ (HSF) cells/well and cultured overnight at 37°C in 5% CO_2_ to make them attach. The supernatants were discarded, and the cells were treated with 1 to 128 μg/ml of CC and 0.3% DMSO for 24 h. CCK-8 reagent (Dojindo, Japan) was added to each well, and the absorbance was recorded at 450 nm after 1–4 h of incubation (Shin et al., 2019). The experiments were performed in triplicate. The results were presented as IC_50_ (the half maximal inhibitory concentration) (Weber et al., 2019). The cell viability was calculated as follows:


$$\mathrm{Viability}\left(\%\right)=\left(1-\frac{{\text{A}}_{0.3\%\,\mathrm{DMSO}}-{\text{A}}_{\text{sample}}}{{\text{A}}_{0.3\%\,\mathrm{DMSO}}}\right)\times 100\%$$

### Skin Abscess Model

The skin abscess model has been approved by the Ethics Committee of the Third Xiangya Hospital of Central South University (N0:2019sydw0211). Seven-week-old female ICR mice (Hunan SJA Laboratory Animal Co., Ltd., China) with an average weight of 25 g was used in this experiment. The mice (*n* = 5) were anesthetized with sodium pentobarbital (50 mg/kg) via intraperitoneal injection. Then 50 μl of *S. aureus* ATCC 43300 of approximately 1.5 × 10^8^ CFU/ml was subcutaneously injected into the dorsum. At 1-h post-infection, 100 μl of CC (5, 10, and 15 mg/kg), 0.1% DMSO (vehicle), and vancomycin (15 mg/kg, positive control) were injected subcutaneously at the same site. The development of abscess was closely monitored daily. On the third day, the abscess was measured, incised, and homogenized in saline to quantify the viable bacterial cells. The tissue surrounding the abscess preserved in formalin was used for hematoxylin and eosin (H&E) staining (Pletzer et al., 2018). The abscess model of *S. aureus* ATCC 43300 persisters induced by 100 × MIC rifampicin was conducted as above. The mice were sacrificed after treatment of 6–8 h, and CFU counting was performed.

### Statistical Analysis

The results were presented as mean ± standard deviation (SD). The statistical analysis was performed by GraphPad Prism 7 (GraphPad Software, San Diego, CA, United States), and the data were analyzed by Student’s *t-*test and one-way analysis of variance, and a *p*-value of < 0.05 was considered significant.

## Results

### Antimicrobial Effects of Candesartan Cilexetil Against *Staphylococcus aureus*

**FIGURE 1:**
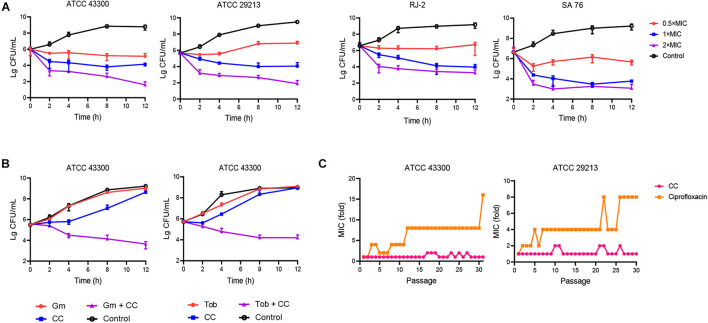
The antibacterial effect of candesartan cilexetil (CC) toward *Staphylococcus aureus*. **(A)** *S. aureus* ATCC 43300, ATCC 29213, RJ-2, and SA 76 (MIC = 16 μg/ml) were exposed to CC at 0.5×, 1×, and 2× MIC; the bacterial abundance changed over time. **(B)** Time-kill assays of *S. aureus* ATCC 43300 treated with 4 μg/ml of CC alone or in combination with 8 μg/ml of gentamicin (left) or 32 μg/ml of tobramycin (right). The results were expressed in CFU/ml. The experiments were conducted in triplicate and presented as mean ± SD. **(C)** A continuous resistance passage of sub-inhibitory concentrations of CC and ciprofloxacin against *S. aureus* ATCC 43300 and ATCC 29213; this procedure was repeated for 31 days.

The antibacterial spectrum of CC and daptomycin against a panel of strains was determined (Table 1). Among them, CC had strong antibacterial potency against type and clinical strains of *S. aureus* with MICs and MBCs of 8–16 and 16–32 μg/ml. Daptomycin was chosen to compare their antibacterial activity as daptomycin was one of the last-resort treatments for MRSA. The MICs and MBCs of daptomycin against *S. aureus* were 0.5–4 and 1–4 μg/ml. However, CC was not active against Gram-negative pathogens with MICs > 64 μg/ml. To study the time-dependent and dose-dependent effect against *S. aureus*, time-kill assays were conducted. CC (2 × MIC) exhibited a strong bactericidal effect against *S. aureus* within 12 h. Treatment with 0.5 × and 1 × MIC of CC inhibited the growth of *S. aureus* (Figure 1A). In keeping with the above results, the OD_630nm_ was not detectable at 0.5 ×, 1 ×, and 2 × MIC (Supplementary Figure 1).

**TABLE 1:** The antimicrobial effect of candesartan cilexetil (CC) against a series of strains.

Strains	CC		Daptomycin	
		
	Minimal inhibitory concentrations (MIC) (μg/ml)	Minimal bactericidal concentrations (MBC) (μg/ml)	MIC (μg/ml)	MBC (μg/ml)
*Staphylococcus aureus* ATCC 25923	8	16	0.5	2
*S. aureus* ATCC 29213	8	16	0.5	1
*S. aureus* ATCC 43300	16	32	0.25	1
*S. aureus* Newman	8	16	4	4
*S. aureus* RJ-2	16	32	2	4
*Staphylococcus epidermidis* ATCC 12228	8	16	0.25	0.5
*Enterococcus faecalis* ATCC 29212	32	64	2	4
*Klebsiella pneumoniae* ATCC 700603	>64	>64	>64	>64
*Pseudomonas aeruginosa* PAO1	>64	>64	>64	>64
*P. aeruginosa* PA47	>64	>64	>64	>64
*Escherichia coli* ATCC 25922	>64	>64	>64	>64
*Acinetobacter baumannii* ATCC 1195	>64	>64	>64	>64

The synergistic antimicrobial activities between CC and antibiotics were evaluated by the checkerboard method. CC increased the susceptibility of *S. aureus* ATCC 43300 toward gentamicin and tobramycin (eightfold reduction in MIC) with both FICI of 0.375, displaying a rather considerable synergistic effect (Table 2). The interaction between CC and amikacin, methicillin and ciprofloxacin (FICIs ≤ 0.75) yielded a partial synergy. The results of the time-kill assays showed that the sub-inhibitory concentrations of tobramycin or gentamicin were ineffective in reducing the cell count. However, when CC was combined with tobramycin or gentamicin, the number of viable cells was reduced by 100- to 1,000-fold within 12 h (Figure 1B).

**TABLE 2:** CC combination with other antibiotics.

Antibacterial agent	MIC_A_ (μg/ml)		MIC_B_ (μg/ml)		FICI	Outcome
		
	Alone	Combination	Alone	Combination		
Tobramycin	256	32	16	4	0.375	Synergism
Gentamicin	64	8	16	4	0.375	Synergism
Amikacin	16	2	16	8	0.625	Partial synergism
Oxacillin	64	16	16	8	0.75	Partial synergism
Ciprofloxacin	0.25	0.0625	16	8	0.75	Partial synergism
Clindamycin	8	4	16	8	1	Additivity
Vancomycin	1	0.5	16	8	1	Additivity

*The checkerboard assay exhibited that CC had synergistic antibacterial effect with gentamicin or tobramycin with a fractional inhibitory concentration index (FICI) of 0.375 against *S. aureus* ATCC 43300. A represents other antibiotics; B represents CC.*

In the continuous passage culture at sub-inhibitory concentrations, although the control antibiotic ciprofloxacin represented 8- to 16-fold shift in MIC against *S. aureus* ATCC 29213 and ATCC 43300 after 31 days of passages, the MIC value of CC remained almost unchanged over the whole process of the study (Figure 1C). As for one step resistance, the spontaneous resistance frequency of 4 × MIC rifampicin and ciprofloxacin against *S. aureus* ATCC 43300 was 4.76 × 10^–9^ (± 1.94 × 10^–9^) and 1.78 × 10^–7^ (± 4.32 × 10^–8^), respectively (Table 3). On other hand, there were almost no mutant colonies on the CC-treated agar. The results showed that CC was superior to rifampicin and ciprofloxacin in reducing *S. aureus* resistance mutation colonies both in the short-term and long-term development of resistance.

**TABLE 3:** Spontaneous resistance frequency of CC for *S. aureus*.

Strains	Antimicrobial	Spontaneous resistance frequency (±SD)	
		
		2 × MIC	4 × MIC
ATCC 43300	CC	<9.52 × 10^–10^ (±1.94 × 10^–10^)	<1.03 × 10^–10^ (±2.24 × 10^–10^)
	Rifampin	1.59 × 10^–8^ (±7.36 × 10^–9^)	4.76 × 10^–9^ (±1.94 × 10^–9^)
	Ciprofloxacin	9.42 × 10^–7^ (±2.19 × 10^–7^)	1.78 × 10^–7^ (±4.32 × 10^–8^)
ATCC 29213	CC	<8.33 × 10^–10^ (±1.70 × 10^–10^)	<9.72 × 10^–10^ (±2.60 × 10^–10^)
	Rifampin	9.72 × 10^–9^ (±3.54 × 10^–9^)	6.94 × 10^–9^ (±5.47 × 10^–9^)
	Ciprofloxacin	2.44 × 10^–7^ (±3.68 × 10^–8^)	9.72 × 10^–9^ (±6.44 × 10^–9^)

### Antibiofilm Activity of Candesartan Cilexetil Against *Staphylococcus aureus* Biofilm

**FIGURE 2:**
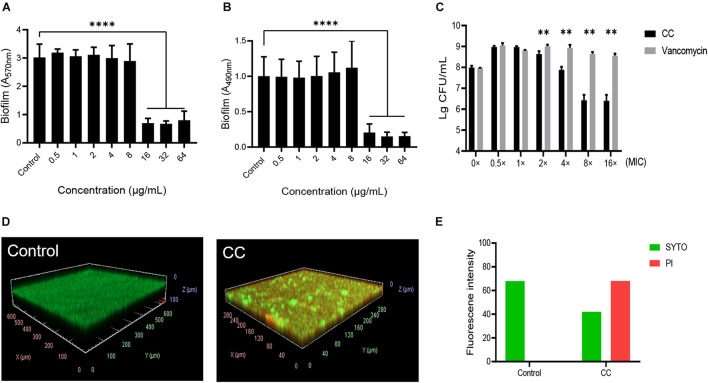
The effect of CC on methicillin-resistant *S. aureus* (MRSA) biofilm. The bacterial suspension of *S. aureus* ATCC 43300 was treated with 0–64 μg/ml of CC for 24 h. **(A)** The biofilm was stained with CV and then measured at A_570nm_. **(B)** The biofilm was stained with XTT and then measured at A_490nm_. **(C)** The bactericidal effect of CC or vancomycin on the preformed biofilm. **p < 0.01, ****p < 0.0001. **(D)** The biofilm was treated with 64 μg/ml CC for 24 h and stained with SYTO9 (green) and PI (red). The images were processed with ImageJ. **(E)** The corresponding fluorescence quantitative result. The proportion of live cells (green) and dead cells (red) decreased and increased significantly after CC treatment. Results were presented as mean ± SD. *p < 0.05.

CV and XTT staining (A_570nm_ and A_490nm_) were used to quantify total biofilm biomass, and a significant reduction was observed when CC was 16 μg/ml (Figures 2A,B). A_570nm_ decreased from 3.02 ± 0.44 to 0.7 ± 0.15 (*t* = 13.95, *p* < 0.05), and A_490nm_ decreased from 1.00 ± 0.26 to 0.21 ± 0.11 (*t* = 7.95, *p* < 0.05), which indicated that CC significantly inhibited the formation of MRSA biofilm. Moreover, CC reduced bacteria amount on preformed biofilm (Figure 2C). CLSM was used to further observe the effect of CC on the mature biofilm morphology. The live and dead cells were dyed green and red, respectively, while using SYTO9 and PI. When CC concentration was 64 μg/ml, compared with control, the proportion of dead cells (red) increased significantly (Figure 2D). Consistent with the results of CLSM, the intensity of green fluorescence decreased, whereas the red fluorescence intensity increased (Figure 2E). Taken together, these results indicated that CC had antibiofilm activity.

### Persister Killing Effects of Candesartan Cilexetil

**FIGURE 3:**
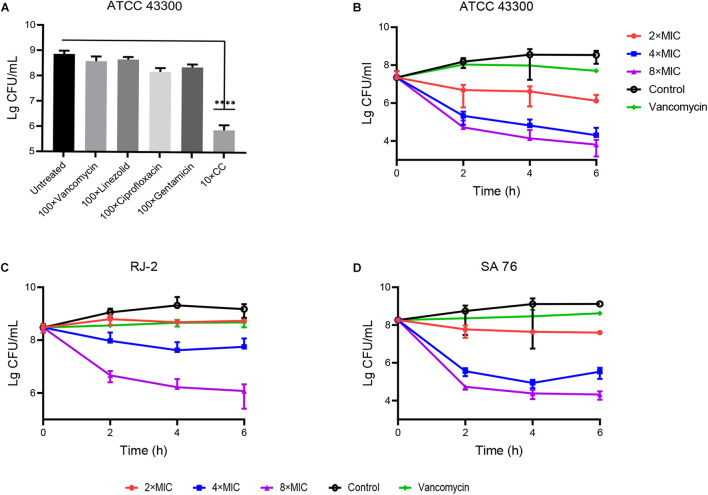
Antibacterial effect of CC on MRSA persisters. **(A)** The stationary-phase *S. aureus* ATCC 43300 persisters were treated with 10× CC and 100× MIC antibiotics for 6 h; no drug as a control. **(B–D)** *S. aureus* ATCC 43300, RJ-2, and SA-76 were treated with 2×, 4×, and 8× MIC CC and removed for serial dilution at 0, 2, 4, and 6 h.

*Staphylococcus aureus* was susceptible to vancomycin and ciprofloxacin. However, the stationary phase cells showed resistance to these antibiotics at 100 × MIC, which indicated that resistance to conventional antibiotics was a common feature of persisters (Fisher et al., 2017). Still, 10 × MIC CC significantly reduced the colony counting of *S. aureus* ATCC 43300 persisters within 6 h compared with conventional antibiotics (Figure 3A). In persister killing assays, 4–8 × MIC CC retained antibacterial activity against MRSA at 6 h by reducing 2–3 log10 persister cells compared with the initial inoculum (Figures 3B–D). The killing effect against persisters induced by rifampicin was also pronounced as shown in Supplementary Figure 2. In contrast, vancomycin was unable to kill MRSA persister cells even at 10 × MIC.

### The Mechanism of Action of Candesartan Cilexetil on *Staphylococcus aureus*

**FIGURE 4:**
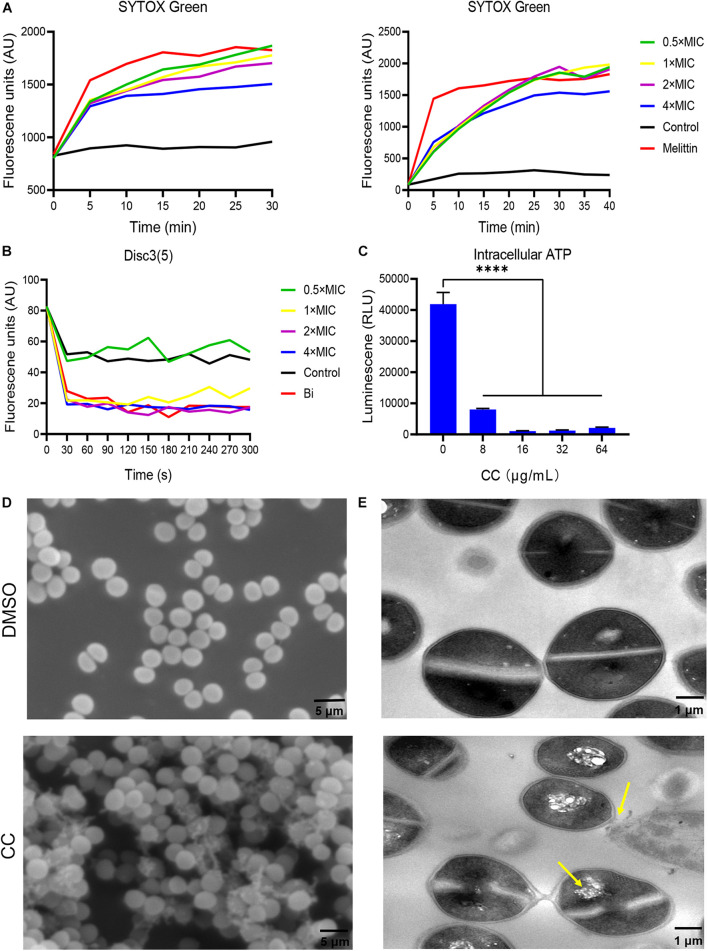
Antibacterial mechanism of CC against *S. aureus* ATCC 43300. **(A)** To treat MRSA planktonic cells (left) and persisters (right), 0.5×, 1×, 2×, and 4× CC were used, and the fluorescence intensity of SYTOX Green changed. Melittin (10 μg/ml) and 0.1% DMSO were used as positive and negative controls. **(B)** The fluorescence intensity change trend of DISC3(5). **(C)** Intracellular ATP level after CC treatment. **(D)** Scanning electron microscopy (SEM) and **(E)** transmission electron microscopy (TEM) were used to observe the cell morphology of MRSA after treatment with CC and DMSO. The yellow arrow points to cell cavities and cell adhesion. Scale bars, 5 μm (left) and 1 μm (right).

Daptomycin is one of last-line treatments targeting MRSA by membrane destruction, which is correspondent with our hypothesis that CC targets bacterial membrane. SYTOX Green is a fluorescence dye for detecting membrane permeability. The fluorescence intensity increases when it is passed through a defected membrane, uptake into bacterial cell, and bound to nucleic acid (Kim E. Y. et al., 2020). In a similar manner, melittin is a widely studied cell membrane disruptor and regarded as a positive control (Takahashi et al., 2013). As expected, in Figure 4A, *S. aureus* ATCC 43300 planktonic and persister cells treated with varying concentrations of CC showed remarkable SYTOX Green staining within 30 min of incubation as melittin, implying virtual membrane damage. MRSA cells incubated without compounds exhibited negligible fluorescent augmentation. DiSC3(5) dye assay reflecting plasma membrane potential change indicated that CC depolarized the bacterial membrane (Figure 4B). To prove the membrane-targeting traits of CC, cellular ATP leakage tests were executed. CC conspicuously reduced intracellular ATP levels in a dose-dependent manner as respected (Figure 4C). Under SEM, the surface morphology of untreated MRSA cells was intact and smooth. MRSA treated by 10 × CC was obviously distorted with an unclear profile, collapsed surface, and membrane disruption (Figure 4D). TEM showed that the bacterial membrane of the treated group was disturbed together with vacuole formation and narrow cytoplasm (Figure 4E). Overall, these results pointed to the fact that CC caused the membrane injury of MRSA and eventually led to the death of bacteria.

### Hemolysis Rate and Cytotoxicity

**FIGURE 5:**
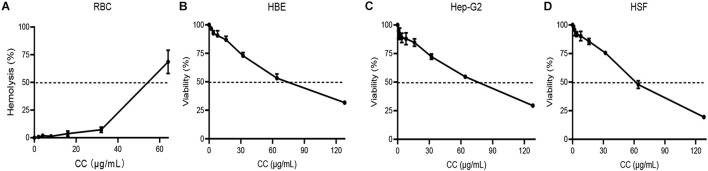
Hemolytic assay and mammalian cytotoxicity assay. **(A)** Red blood cells (RBCs) were treated with 0–64 μg/ml of CC with Tritonx-100 as positive control. **(B–D)** Three cell lines, including HBE, Hep-G2, and HSF, were treated with 0–128 μg/ml of CC. Their IC_50_ was > 50 μg/ml.

In RBC hemolytic test, the HC_50_ was 54.34 μg/ml (Figure 5A and Supplementary Table 1). The viability of HBE, Hep-G2, and HSF were inhibited by CC with IC_50_ of 70.36, 69.01, and 58.22 μg/ml, respectively (Figures 5B–D). The IC_50_ and HC_50_ values of CC were higher than its MICs and MBCs, showing the possibility that the antibacterial concentration *in vivo* could be potentially safe to apply for *S. aureus*.

### Effective Antimicrobial Effects of CC *in vivo*

**FIGURE 6:**
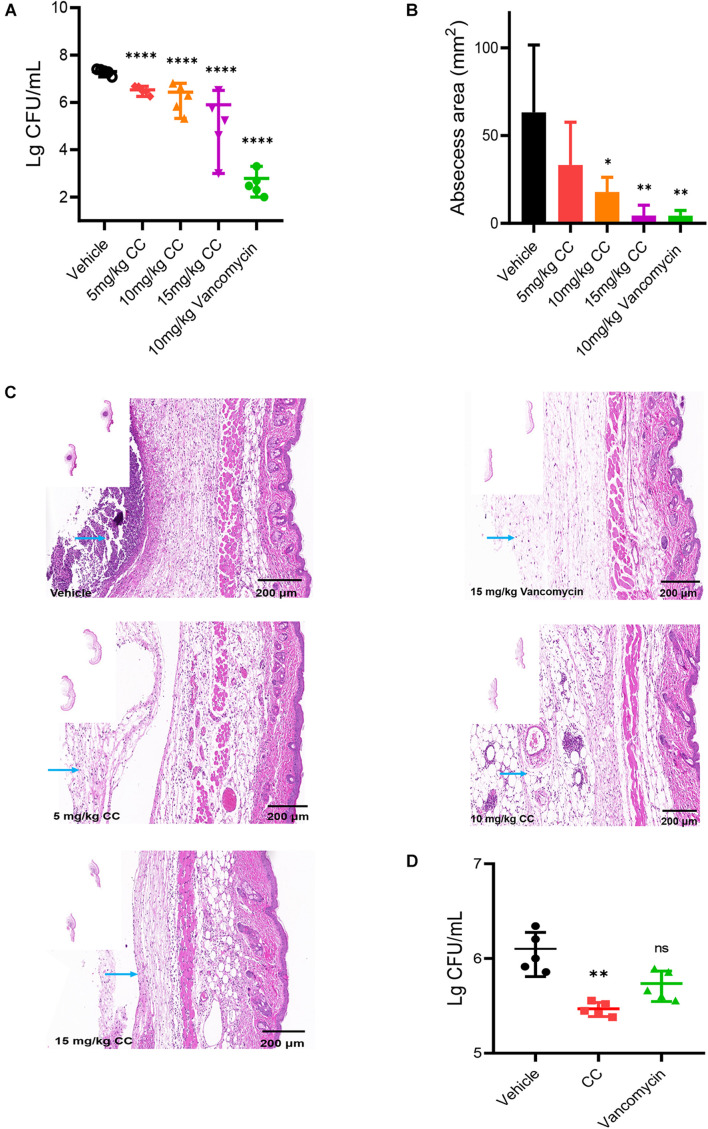
The effect of CC *in vivo* model. **(A)** Female mice (*n* = 5) was subcutaneously injected with 7.5 × 10^6^ CFU/ml of *S. aureus* ATCC 43300 and treated with DMSO, 5, 10, and 15 mg/kg of CC, and 15 mg/kg of vancomycin. The results were expressed as CFU/ml. *p < 0.05, **p < 0.01, ****p < 0.0001. **(B)** The abscess area was measured. **(C)** Hematoxylin and eosin (H&E) staining (7×) showed that the histopathological change (abscess area and inflammatory cells) was significantly reduced after treatment. Scale bars, 200 μm. The blue arrow points to inflammatory cells. **(D)** Female mice (*n* = 5) were injected subcutaneously with approximately 7.5 × 10^6^ CFU/ml of *S. aureus* ATCC 43300 persisters and treated with DMSO, 30 mg/kg of CC, and 15 mg/kg of vancomycin.

The skin abscess model was performed to evaluate the potential therapeutic effect of CC *in vivo.* As a positive control, vancomycin significantly reduced the bacterial abundance more than 1,000-fold and the area of abscesses (*p* < 0.05) (Figures 6A,B). Compared with the vehicle group, a reduction in average bacterial load approximately six-fold, seven-fold, and 25-fold were observed when treated with 5, 10, and 15 mg/kg of CC, respectively (*p* < 0.0001). Consistent with the above results, histopathological analysis showed that after CC treatment, the inflammation of the skin abscess was significantly reduced (Figure 6C). As for MRSA persisters, 30 mg/kg of CC led to an approximately fourfold decrease (*p <* 0.01) (Figure 6D). The levels of inflammatory cytokines IL-1β, IFN-γ, and TNF-α were determined. As shown in Supplementary Figure 3, the IL-1β and IFN-γ levels made no significant difference after treatment with CC compared with those of the control group (*p* > 0.05). Though the level of TNF-α was lower than the control group, there was no distinct difference in quantity. These results proved that CC might present a potential therapy for *S. aureus*-infected diseases caused by antibiotic-resistant or persisters cells.

## Discussion

Rapid emergence of antibiotic-resistant bacteria to conventional antimicrobials leads to failure of traditional therapies to counter for bacterial infections (Kim W. et al., 2020). Therefore, new anti-infective agents should be introduced to bypass the shortcomings. As an FDA-approved drug, CC exhibited an antibacterial effect against Gram-positive bacteria especially MRSA with a low possibility of drug resistance, which might play a crucial role in the treatment of infections caused by *S. aureus.*

The conventional antibiotics face difficulties as effective therapeutic guidelines for biofilm-associated infections with vancomycin is of no exception (Zhang et al., 2019). Through CV and XTT staining assay, CC was found effectively to prevent biofilm formation. Moreover, microorganisms within mature biofilm treated with high concentrations of CC were reduced; nevertheless, the extracellular polymer secreted by the remaining bacteria caused no significant reduction in the biomass of the biofilm.

As already reported in the literature, new antimicrobial candidates that target bacterial cell membranes, including PQ401, CD437, and bithionol, etc., have been identified. Membrane-targeting molecules are able to penetrate into the membranes of bacteria and enter the cell due to the simple lipid layers of *S. aureus* cell membranes (Yuan et al., 2019). CC was able to compromise MRSA membrane integrity in a concentration-dependent manner. Due to membrane damage, antimicrobials currently generate a rapid loss of substances with low molecular weight (MW) from within the cell, for instance, ATP (MW 0.5 kDa). We actually found that CC reduced intracellular ATP levels. The results of SEM and TEM further confirmed that CC caused membrane damage, noticeable leakage of intracellular contents, and even the loss of bacterial structure (Figure 4). All the experiments supported our assumption that CC was a membrane-active agent and exhibited prominent properties such as rapid bactericidal effect, low possibility of bacterial resistance, and antipersister activity.

The antibacterial mechanism of most antibiotics is achieved through the influence of the structure and function of bacterial macromolecules (nucleic acids, lipids, carbohydrates, and proteins) (Zhang et al., 2019). Bacterial persisters are a subpopulation of cells that exhibit metabolically inactive state and antibiotic tolerance. Vancomycin, linezolid, and ciprofloxacin are commonly used for treating *S. aureus*-associated infections, yet their chemotherapy effect on persister processes is ineffective. CC was capable of killing persisters quickly due to its rapid membrane penetration, which is consistent with the characteristics of membrane-targeting antimicrobial agents. Gentamicin is an aminoglycoside antibiotic that binds to bacterial ribosomal subunits to inhibit protein synthesis (Tsai et al., 2013). In the field of antimicrobial application, the pathogenic microorganisms undergoing treatment with the combination therapy not only improve the efficacy of the anti-infective drugs but also reduce side effects and cytotoxicity. Studies have acknowledged that membrane-targeting agents synergize with aminoglycoside antibiotics against MRSA (Yarlagadda et al., 2020). CC was found to exert a synergetic effect with gentamicin and tobramycin via the checkerboard dilution method. The possible mechanism of action was that sufficient accumulation of CC on the membrane formed a prerequisite for the diffusion of antibiotics into the bacterial cell, thereby inhibiting the synthesis of ribosomal subunit proteins.

To our knowledge, no acute lethal toxicity was found in mice, rats, or dogs after a single oral dose of 2,000 mg/kg of CC. Toxicological experiments showed that CC had no carcinogenic, teratogenic, and mutagenic effects (Nishikawa et al., 1997). In combination with the results that the HC_50_ and IC_50_ were higher than its MIC or MBC (Supplementary Table 1), it was concluded that CC possessed limited hemolysis rate and cytotoxicity. Further exploration to improve its antibacterial activity and toxicity might be achieved by drug combination and chemical modification.

As far as we know, there was limited research on the efficacy of CC in the subcutaneous abscess model. In the MRSA infection model, a topical infection model was chosen to evaluate the treatment effect of CC *in vivo* for the reason that *S. aureus* was the common pathogen of skin infection. In our study, CC reduced the bacterial abundance in topical skin without acute damage in mice model. Previous studies reported that angiotensin-converting enzyme (ACE) inhibitors were able to inhibit the expression of the inflammatory factor tumor necrosis factor-alpha (TNF-α) (Aliper et al., 2017). The determination of cytokines showed that CC would not induce inflammatory response in RAW264.7. This leads us to speculate that CC may exert an important role in anti-inflammatory response *in vivo*. These assays showed that CC exhibited antibacterial and antipersister effect *in vivo* as well.

In summary, CC displayed strong antibacterial activity against multidrug-resistant and -persistent *S. aureus*. Moreover, CC had limited cytotoxicity and low possibility of drug resistance. In a subcutaneous abscess infection model, CC was effective in reducing bacterial burden. These findings highlight the possibility of CC for treating MRSA infection.

## Data Availability Statement

The original contributions presented in the study are included in the article/Supplementary Material, further inquiries can be directed to the corresponding author.

## Ethics Statement

The animal study was reviewed and approved by the Ethics Committee of the Third Xiangya Hospital of Central South University.

## Author Contributions

LX and PS designed all the experiments of this study. LX conducted most of the experiments and wrote the manuscript. LC and SL made data curation and figures. LZ was responsible for purchasing the reagents and materials. ZH and YL were responsible for the accounts and helped complete the supporting experiments. YW supervised the writing process and all the study. All authors contributed to the article and approved the submitted version.

## Conflict of Interest

The authors declare that the research was conducted in the absence of any commercial or financial relationships that could be construed as a potential conflict of interest.

## Publisher’s Note

All claims expressed in this article are solely those of the authors and do not necessarily represent those of their affiliated organizations, or those of the publisher, the editors and the reviewers. Any product that may be evaluated in this article, or claim that may be made by its manufacturer, is not guaranteed or endorsed by the publisher.
