# Supplementary material for: Repurposing Candesartan Cilexetil as Antibacterial Agent for MRSA Infection
Source: Front Microbiol. 2021 Sep 13;12:688772. doi: 10.3389/fmicb.2021.688772 (PMC8473943; doi:10.3389/fmicb.2021.688772)
Supplement: Supplementary file 1 [file Data_Sheet_1.ZIP › Supplementary_Material/Supplementary_Material.docx]

Supplementary Material

**Supplementary methods**

**Persisters Killing Assay Induced by Rifampicin**

*S. aureus* ATCC 43300 were cultured to the stationary phase, then 100 × MIC rifampin were added to continue shaking overnight. The persisters were collected by centrifugation and washing. The cultures were adjusted to OD_630_ = 0.2, different concentrations of CC were added. The viable cells counting were conducted at 6 h. The experiments were conducted three times.

**Cytokines Determination**

## Macrophages RAW264.7 were treated with or without CC. The supernatants collected from macrophages were detected for cytokines determination (IL-1β, IFN-γ and TNF-α) using ELISA kits (Abcam Inc. USA) according to manufacturer's instructions (She et al., 2020).

**Supplementary figures and tables**

**Table S1**. Comparison chart of IC_50_ and HC_50_ with MIC and MBC

|  | IC_50_ | | | HC_50_ | MIC | MBC |
| --- | --- | --- | --- | --- | --- | --- |
| Cells strains | HBE | Hep-G2 | HSF | RBCs | *S. aureus* | *S. aureus* |
| μg/mL | 70.36 | 69.01 | 58.22 | 54.34 | 8～16 | 16～32 |

HBE: human bronchial epithelial cells; HepG2: Human liver cancer cell lines; HSF: human skin fibroblast (HSF) cell lines.

**Figure S1.** The OD630 of CC against *S. aureus* ATCC 43300 and 29213. (A-B), the OD_630_ change of *S. aureus* ATCC 43300 and 29213 exposed to different concentrations of CC, the trend was consistent with time-killing assays.


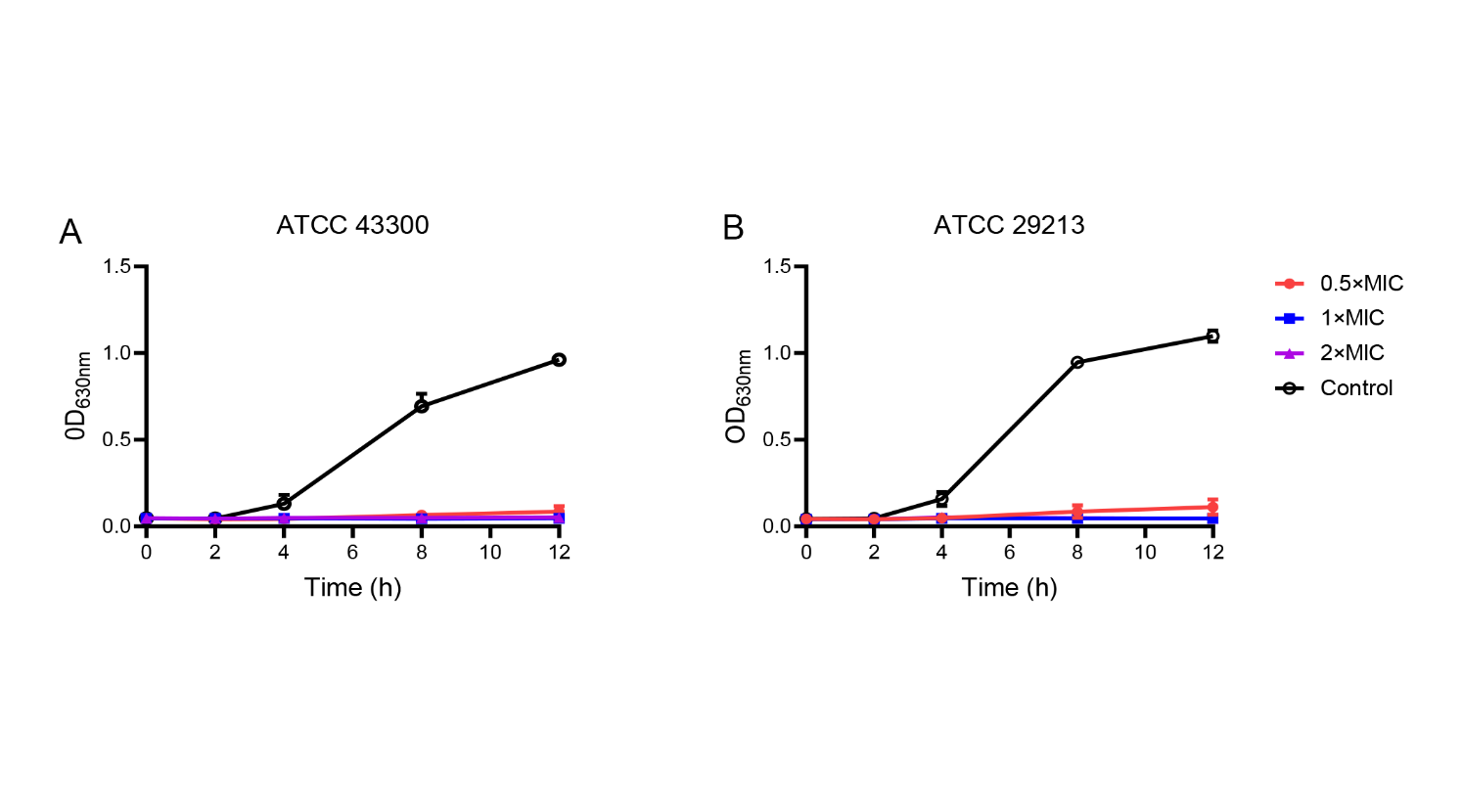


**Figure S2.** The killing effect of CC against *S. aureus* ATCC 43300 persisters induced by rifampicin.


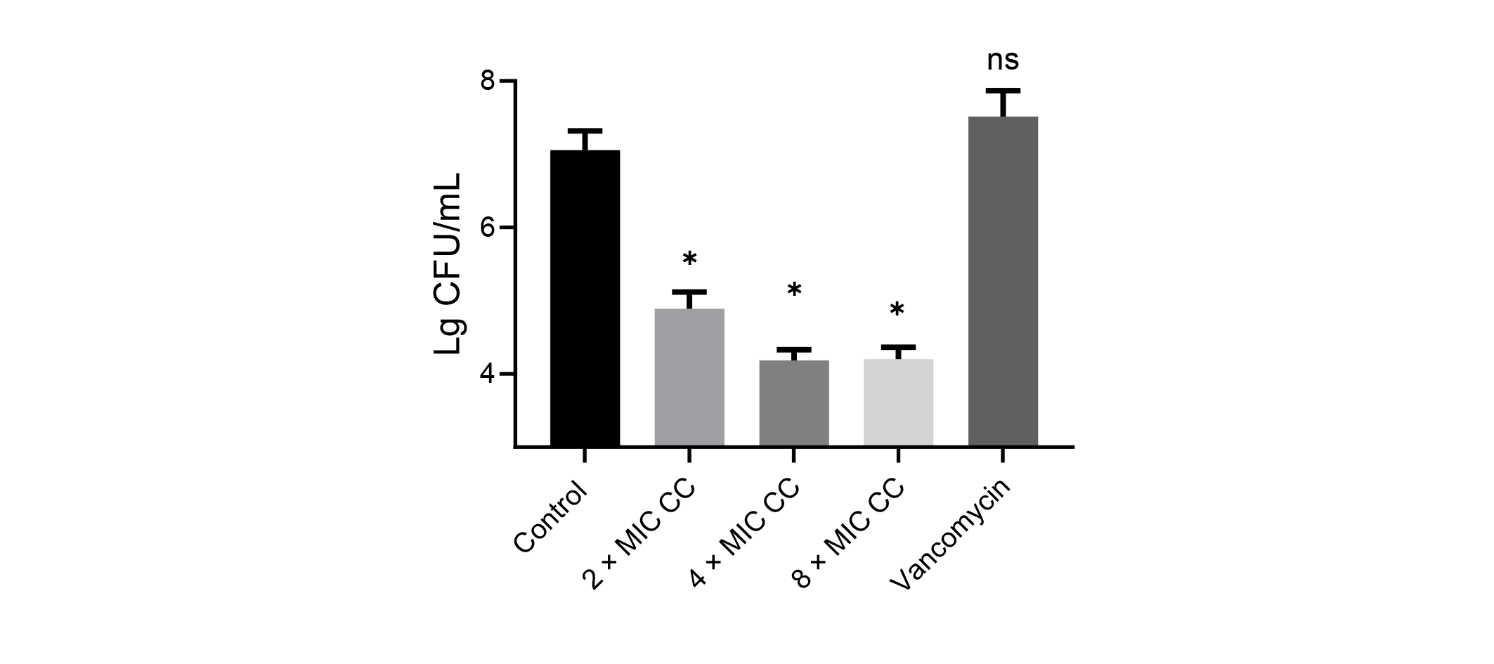


**Figure S3.** Production of the cytokines detected by ELISA.The concentration of CC was 16 μg/mL.

**
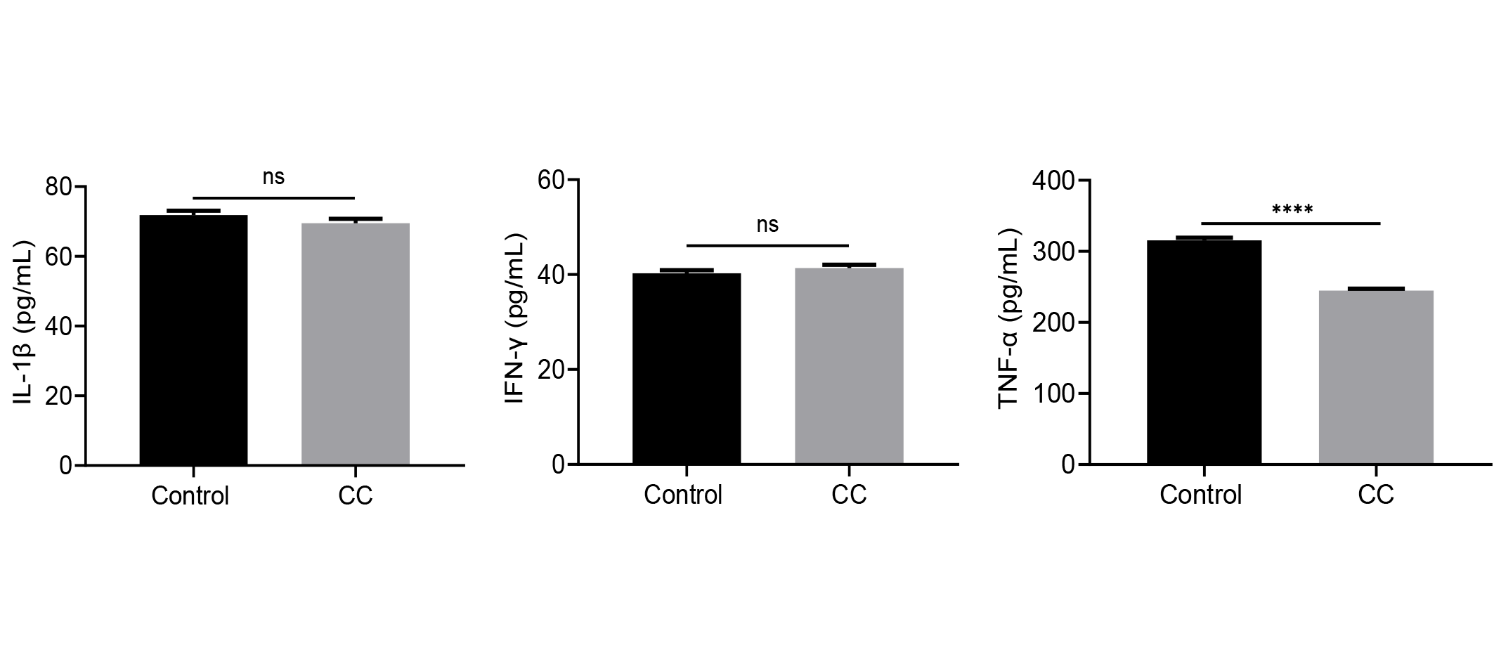
**

**References**

She, P., Liu, Y., Luo, Z., Chen, L., Zhou, L., Hussain, Z., et al. (2020). PA2146 Gene Knockout Is Associated With Pseudomonas aeruginosa Pathogenicity in Macrophage and Host Immune Response. *Front Cell Infect Microbiol* 10**,** 559803. doi: 10.3389/fcimb.2020.559803.
